# The importance of fracture toughness in ultrafine and nanocrystalline bulk materials

**DOI:** 10.1080/21663831.2016.1166403

**Published:** 2016-04-12

**Authors:** R. Pippan, A. Hohenwarter

**Affiliations:** ^a^ Erich Schmid Institute of Materials Science, Austrian Academy of Sciences, Leoben, Austria; ^b^ Department of Materials Physics, Montanuniversität Leoben, Leoben, Austria

**Keywords:** Fracture toughness, severe plastic deformation, ultrafine-grained, nanocrystalline, anisotropy

## Abstract

The suitability of high-strength ultrafine and nanocrystalline materials processed by severe plastic deformation methods and aimed to be used for structural applications will strongly depend on their resistance against crack growth. In this contribution some general available findings on the damage tolerance of this material class will be summarized. Particularly, the occurrence of a pronounced fracture anisotropy will be in the center of discussion. In addition, the great potential of this generated anisotropy to obtain high-strength materials with exceptionally high fracture toughness in specific loading and crack growth directions will be enlightened.

**IMPACT STATEMENT**

Severely plastically deformed materials are reviewed in light of their damage tolerance. The frequently observed toughness anisotropy allows unprecedented fracture toughness – strength combinations.

## Introduction

In engineering, Young’s modulus, strength, ductility and fracture toughness are the most important mechanical properties for the proper mechanical design of structural components. To increase the strength in terms of the yield strength, *σ_y_*, and ultimate strength, *σ*_UTS_, of metallic materials, different strengthening mechanisms are known. Grain refinement has shown to be a very efficient method, especially when the grain size is reduced below one micron into the ultrafine-grained (UFG) or nanocrystalline (NC) regime.[1–4] This strengthening mechanism has been extensively investigated in the last decades and besides the improvement of strength special attention has been devoted to the change in ductility. General observations have been that below a certain critical grain size, the strain at uniform elongation decreases to relatively small values and therefore also the total fracture strain. Other ductility-related measures such as the reduction in area and the true fracture strain show the same decreasing tendency. The deterioration can be widely associated with the decrease of work hardening capacity. A vast number of studies are devoted to this conflict between strength and ductility and to strategies to mitigate the drawback.[5,6] Compared to this large body of research, relatively less attention is devoted to the change of fracture toughness when the grain size is reduced to the UFG or NC regime.

**Figure 1.**
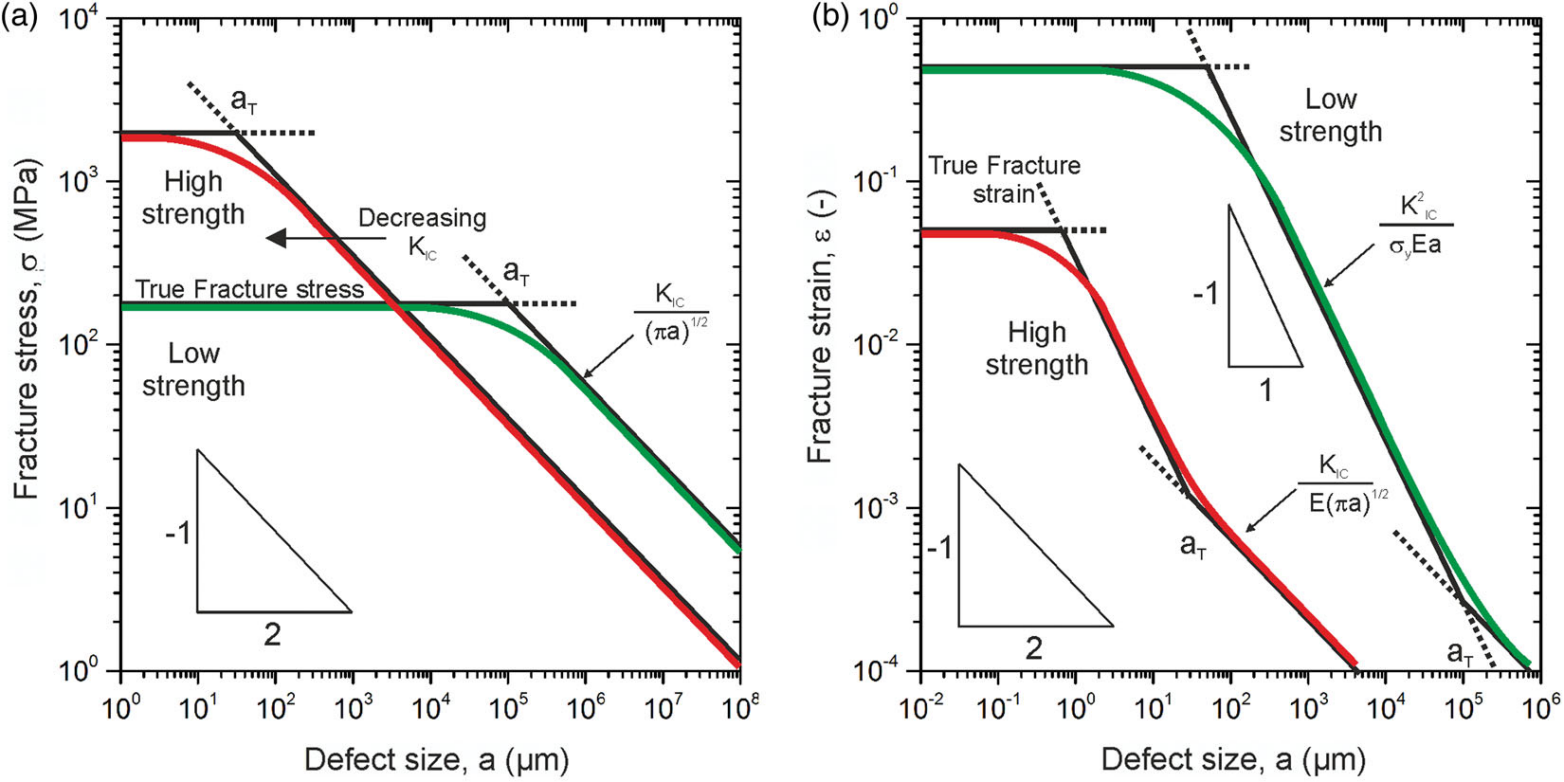
Static Kitagawa–Takahashi plots demonstrating the importance of defect-sensitive design. (a) Comparison between a low- and high-strength material in a stress-based plot. (b) Adapted Kitagawa–Takahashi diagram depicting the influence of fracture toughness and crack length on the fracture strain in a comparison between a low- and high-strength material.

However, fracture toughness, for example expressed with the critical stress intensity *K*_IC_, would be even more important to examine in high-strength, including therefore also UFG and NC materials, than in low-strength materials, which is schematically demonstrated with Figure 1. This plot is a static Kitagawa–Takahashi diagram [7] where the fracture strength, *σ*_fr_, Figure 1(a), or fracture stain, *ε*_fr_, Figure 1(b), of a large tensile sample is plotted as a function of the size of a crack like defect, *a*. Focusing first on the fracture stress, for very short cracks the fracture stress is independent of the crack length. For longer cracks there is a transition from the crack length-independent to a crack length-dependent failure regime. For defect sizes or crack lengths larger than 2 or 3 times *a*_T_, where
(1) 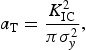

the fracture strength is controlled by the fracture toughness, *K*_IC_, and the crack length, *a*. Only for defect sizes somewhat smaller than *a*_T_ the fracture stress is solely governed by the strength of the material. In other words only for this case the ‘strength-capacity’ of the material can be fully used. For simplicity, let us first assume that the fracture toughness of a high-strength material is equal to the fracture toughness of the low-strength material, which is normally rather unlikely. For high-strength materials the transition length *a*_T_, which also delineates the linear elastic from the elastoplastic fracture mechanics regime, becomes significantly smaller, see Figure 1(a), because of its inverse proportionality to 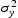. Furthermore, one should take into account that in high-strength materials the fracture toughness is usually smaller shifting the transition to even smaller transition lengths as shown in Figure 1. The transition length *a*_T_ for a typical high-strength material with *σ_y_* = 2,000 MPa and a realistic fracture toughness, *K*_IC_, of about 20 MPa m^1/2^ is only ∼30 µm whereas for a low-strength material (*σ_y_* = 180 MPa) and a high fracture toughness of typically 100 MPa m^1/2^ *a*_T_ is about 100 mm. This stress-based consideration illustrates the importance of fracture toughness in high-strength ultrafine and NC materials.

Even though for defect sizes smaller than *a*_T_ the fracture stress controls failure, the fracture toughness is still a very important parameter, when the resulting fracture strain, which is a measure for ductility in a specimen with a natural defect, is examined. In the regime of the elastoplastic fracture mechanics (defect sizes smaller than *a*_T_) the fracture strain is governed by fracture toughness and yield stress, see Figure 1(b). The fracture strain in this regime is proportional to the square of the fracture toughness, and inversely proportional to the yield strength and crack length:
(2) 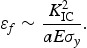



This indicates the enormous importance of the fracture toughness even in the case of large-scale yielding, where the fracture load is dominated by the strength, but the fracture strain, that is ductility, is governed by the fracture toughness and the yield strength. Only in the plateau-regime, below the transition length, the intrinsic fracture strain is decisive. Then, size and distribution of remaining inclusions, the void size evolution during deformation and the hardening behavior are the main factors controlling the fracture strain.

Despite the enormous importance of the fracture toughness in high-strength NC and UFG bulk materials, there are only a few research groups that have been dealing with this aspect experimentally.[8–12] One of the reasons is that many of the syntheses techniques used to generate NC materials can produce only small quantities or very thin layers. The determination of the fracture toughness in such cases is experimentally difficult, becomes sample size dependent or the used approaches are only applicable to very brittle materials.[13,14] Severe plastic deformation (SPD) offers the production of relatively large quantities of UFG and NC materials in bulk form. However, even for this class of materials there are only few studies focusing on the fracture toughness.[15–18] One of the main results of these studies was that the fracture toughness in these materials is very sensitive to the grain shape. This finding is quite significant because many SPD processes deliver elongated and, only very rarely, equiaxed microstructures in all three sample dimensions.

The goal of this review is to show that the generated anisotropy can be used to obtain high-strength materials with exceptionally high fracture toughness, however, only for specific loading and crack growth directions. In the following sections different examples demonstrating this fracture toughness anisotropy are presented and in last section the reasons for this behavior and the consequences for future material design are discussed.

## Fracture behavior of SPD materials: some general observations

One of the first studies directly focusing on the influence of the testing direction on fracture toughness was performed with iron [16] and nickel [17]. Even though these materials differ markedly, as far as crystal plasticity is concerned, after SPD performed by high pressure torsion (HPT), the saturation microstructure of both materials is fairly similar. The grain size is around 200–300 nm depending on the viewing direction with a hardness of 380 HV for nickel and 420 HV for iron, and ultimate strengths of 1,400 MPa and 1,600 MPa, respectively.

**Figure 2.**
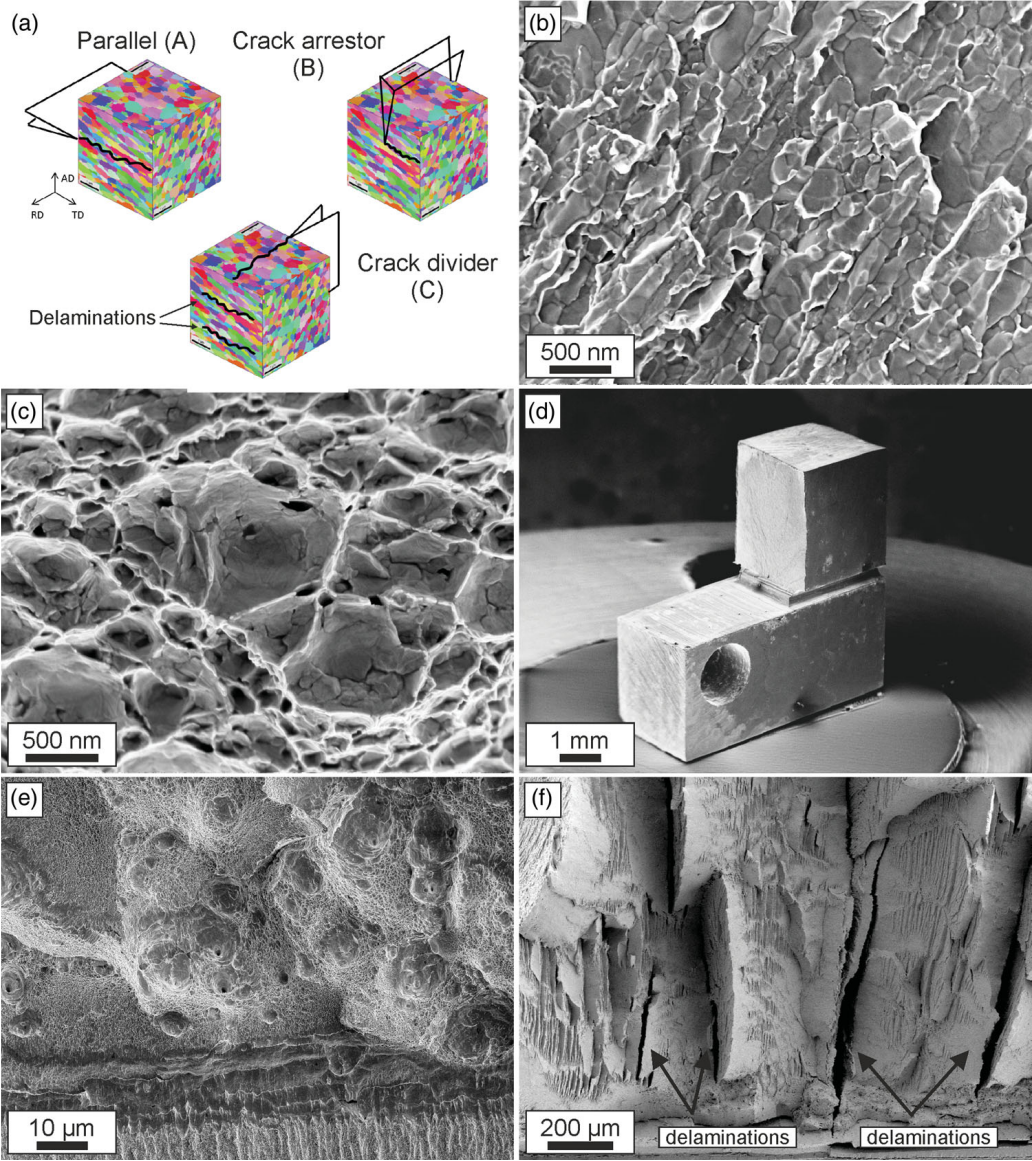
Overview describing the fracture behavior of UFG-iron and nickel. (a) Principal crack planes and crack growth directions investigated for both materials. For simplicity, the crack plane and crack propagation direction of a specimen orientation are abbreviated as A, B or C. Fractographs for crack growth along the elongated microstructure in iron with intergranular fracture (b) and nickel exhibiting transgranular micro-ductile fracture (c). (d) Iron fracture sample with crack-arrestor orientation. (e) Micro-ductile fracture surface found in Ni for the third testing direction. (f) Fracture surface exhibiting various delaminations (some of them are indicated with arrows) typical for iron for the third orientation (crack-divider orientation).

An important feature of SPD-processed materials is a pronounced alignment and elongation of the microstructure into the principal deformation direction, which is not an exclusive feature of HPT structures but also occurring in other processes such as Accumulative Roll Bonding (ARB) [19] or Equal Channel Angular Pressing (ECAP).[20] An example for these rather anisotropic structures is presented in Figure 2(a) with nickel deformed by HPT exhibiting elongated grains parallel to the tangential direction (TD), looking into the radial direction (RD) and also into the axial direction (AD), however, less pronounced. Parallel to the TD, the microstructure exhibits a relatively equiaxed structure. When performing fracture experiments in the principal possible propagation directions, which are indicated in Figure 2(a), distinctive differences in the resulting fracture toughness combined with extreme variations of the fractography were found, see Figure 2(b)–(f) and Table 1.

**Table 1.** Comparison of fracture toughness in UFG-iron and UFG-nickel. The results of Ni were re-calculated into equivalent critical stress intensities derived from crack tip opening displacement measurements [17].

Iron	*K*_IC_ (MPa m^1/2^)	Nickel	*K*_IC_ (MPa m^1/2^)
A	14.2	A	63.2
B	36.2	B	108.1
C	49.0	C	72.3

Parallel to the grain alignment, UFG-iron exhibits brittle behavior with intergranular fracture, Figure 2(b). The fracture toughness is with ∼14 MPa m^1/2^ lower compared to CG-iron,[21] however, substantially higher than expected from a pure de-cohesion process at the grain boundaries. This means that a distinctive amount of plasticity must be involved in the fracture process. In contrast, in the same testing direction UFG-nickel having a comparable microstructure fails by classical micro-ductile fracture with typical voids in the size range of several grain diameters with a higher fracture toughness in the range of 60 MPa m^1/2^, see Figure 2(c). In the testing direction perpendicular to the long axis of the grains, Figure 2(a), both materials exhibit a fairly high fracture toughness (Table 1), combined with a marked crack deflection into the direction parallel to the long axis of the grains. This configuration can also be named as the crack-arrestor orientation and a typical example for the global crack deflection is presented in Figure 2(d) with an iron sample.

In the third testing direction the grains are also oriented with their long axis perpendicular to the crack propagation direction but the crack runs into the RD instead of the TD (Figure 2(a)). Both materials have an exceptionally high fracture toughness (Table 1) and at the same time high strength. For Ni the fractograph is comparable to the parallel orientation (Figure 2(e)), showing again dimple fracture consisting of large dimples initiated at nonmetallic inclusions and smaller ones between them as presented in Figure 2(c). In contrast, iron exhibits a significant feature on the fracture surface with secondary crack, called delaminations, propagating perpendicular into the primary crack plane and dividing the sample locally into thinner ligaments (Figure 2(f)). For this reason, when testing in this direction such delaminations occur, it is often named crack-divider orientation.^1^ It is important to note that the crack plane of the initiation points of the delaminations near the primary crack tip is the same as the one having samples with the parallel orientation showing an extremely low fracture toughness.

## Micromechanisms of fracture in different grain size regimes

**Figure 3.**
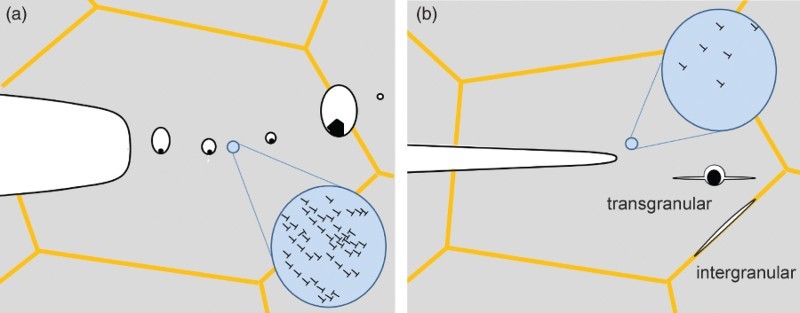
Principal failure types in coarse-grained metals. (a) Micro-ductile fracture through the coalescence of individual voids. (b) De-cohesion process leading to inter- or transgranular fracture. In both cases plasticity, illustrated by the dislocation bundles, is involved.

The micromechanisms controlling the fracture toughness of pure metals and alloys can be divided into two main classes: micro-ductile crack propagation and crack propagation by de-cohesion processes. The principles are depicted in Figure 3. The stages of micro-ductile crack propagation are crack tip blunting by plastic deformation, void formation, often initiated at nonmetallic inclusions followed by void formation at precipitates or interaction of localized shear bands, growth of voids and final coalescence of voids with the blunted crack tip, Figure 3(a). All these phenomena are coupled with local intense plastic deformation.

Crack propagation by de-cohesion of grain and phase boundaries or by cleaving of grains along certain crystallographic planes in metals and alloys is usually associated with local plastic deformation.[22] The typical stages of this brittle crack propagation are blunting of the crack tip by plastic deformation, generation of microcracks, coalescence of these microcracks with the main crack and final fracture of the remaining ligament bridges, Figure 3(b). These cracks can propagate within the grains, transgranular (transcrystalline) or intergranular (intercrystalline).

There is a large variation of these processes which depend on the microstructure and environmental conditions such as temperature or medium and loading conditions (quasi-static, cyclic, pure Mode-I or Mixed-Mode). The complexity of the interaction between the different mechanisms involved in the crack propagation process with the influencing variables is the main reason behind the problems in an unambiguous prediction of the fracture toughness even in the case of classical microcrystalline metals and alloys.[23,24]

What are now the essential differences with respect to crack propagation processes in NC and UFG materials? Micro-ductile and de-cohesion are still the main fracture mechanisms as shown with the presented examples; however, de-cohesion by cleavage of grains (transgranular fracture) seems to disappear and intergranular fracture prevails. In coarse-grained materials, the microstructural features such as grain size or distances between nonmetallic inclusions are large compared to the typical dislocation spacing and the characteristic dimensions of dislocation structures in the plastically deformed zone of a propagating crack. The same is true for the resulting characteristic dimensions of the fracture surface features, that is, spacing and size of voids, size of cleavage planes, which are again large compared to the typical dimensions of the dislocation structures.

For materials with nanometer grains, the situation is different:

- There are only a few dislocations in the interior of the grains, even in UFG and NC materials generated by SPD most of the dislocations are arranged in the vicinity of the grain boundary.[25]
- The density of grain boundaries and grain boundary triple junction is very large, which are initiation sites for pore formation or the generation of nanocracks by de-cohesion.[26–28]
- Precipitates or second phases are usually not in the interior of a grain, they form at grain boundaries or triple junctions.[29,30]
- An essential finding of the fracture toughness investigation of body-centered cubic (bcc) metals is that transgranular crack propagation does not occur below a critical grain size (few 100 nm).[31] A reason for this behavior might be that there is no sufficient space to form the necessary dislocation pile-ups or there are always sufficient boundaries where de-cohesion is easier than the cleavage of the grains.

Consequently, there is a large number of potential places for the formation of pores, nanocracks or nanocrack extension by de-cohesion of grain boundaries or triple junctions to occur. Therefore, it is evident that contrary to microcrystalline metals and alloys besides the grain size, the grain shape plays a dominant role independent of the crack propagation mechanisms by micro-ductile or de-cohesion failure. NC and UFG materials processed by SPD methods always exhibit a more or less pronounced grain shape anisotropy (shape texture) with an alignment of the grains in a certain direction which is a consequence of the synthesis processes. This alignment results in an orientation dependence of the fracture toughness. Similar orientation dependencies are observed in standard coarse-grained engineering alloys; however, in this case the alignment and shape texture of the nonmetallic inclusions are mainly responsible for the orientation dependencies.[32,33] Based on the previously presented iron and nickel results, two classes of NC and UFG materials with distinctively different crack propagation mechanisms can be defined, which will be in the following named as the ‘ductile’ and ‘brittle’ type.

**Figure 4.**
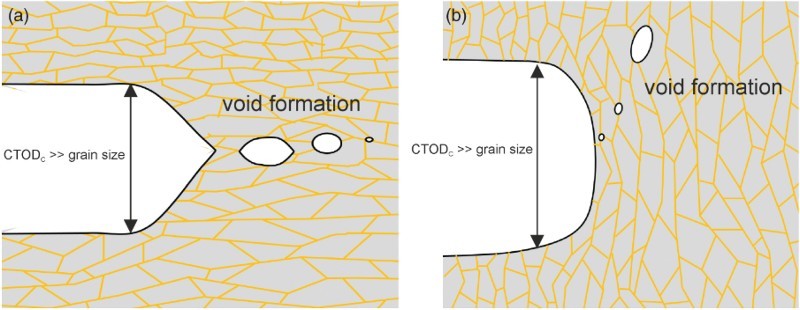
Ductile failure type in NC and UFG metals. (a) Micro-ductile fracture through the coalescence of individual voids. (b) Crack branching into the direction of grain alignment with higher fracture toughness and micro-ductile fracture.

The first group of NC and UFG metals shows micro-ductile crack propagation for all crack propagation directions, however, with different fracture toughness values and often with a pronounced tendency to crack branching into the crack propagation direction parallel to the grain elongation (Figure 2(a) with orientation A–C). The critical crack tip opening displacement, CTOD_c_, before the coalescence of the blunted crack tip with the micro- and nano-pores takes place, is typically between a few and 100 times the grain size, Figure 4(a). The reason for this huge difference in CTOD_c_ with respect to the grain size in the different UFG and NC materials is yet not well understood. In contrast, the observed orientation dependence of CTOD_c_ in the individual testing directions is not surprising. The coalescence of the pores and maybe also the initiation of the pores is governed by plastic deformation and by grain boundary de-cohesion. This should be energetically easier when the majority of grain boundaries are aligned parallel to the pre-crack than perpendicular to it, leading to the crack bifurcation (see Figure 4(b) and Figure 2(a) with orientation B). This type of fracture behavior has been found in various face-centered cubic metals [15,17] but also in bcc metals such as vanadium.[34]

**Figure 5.**
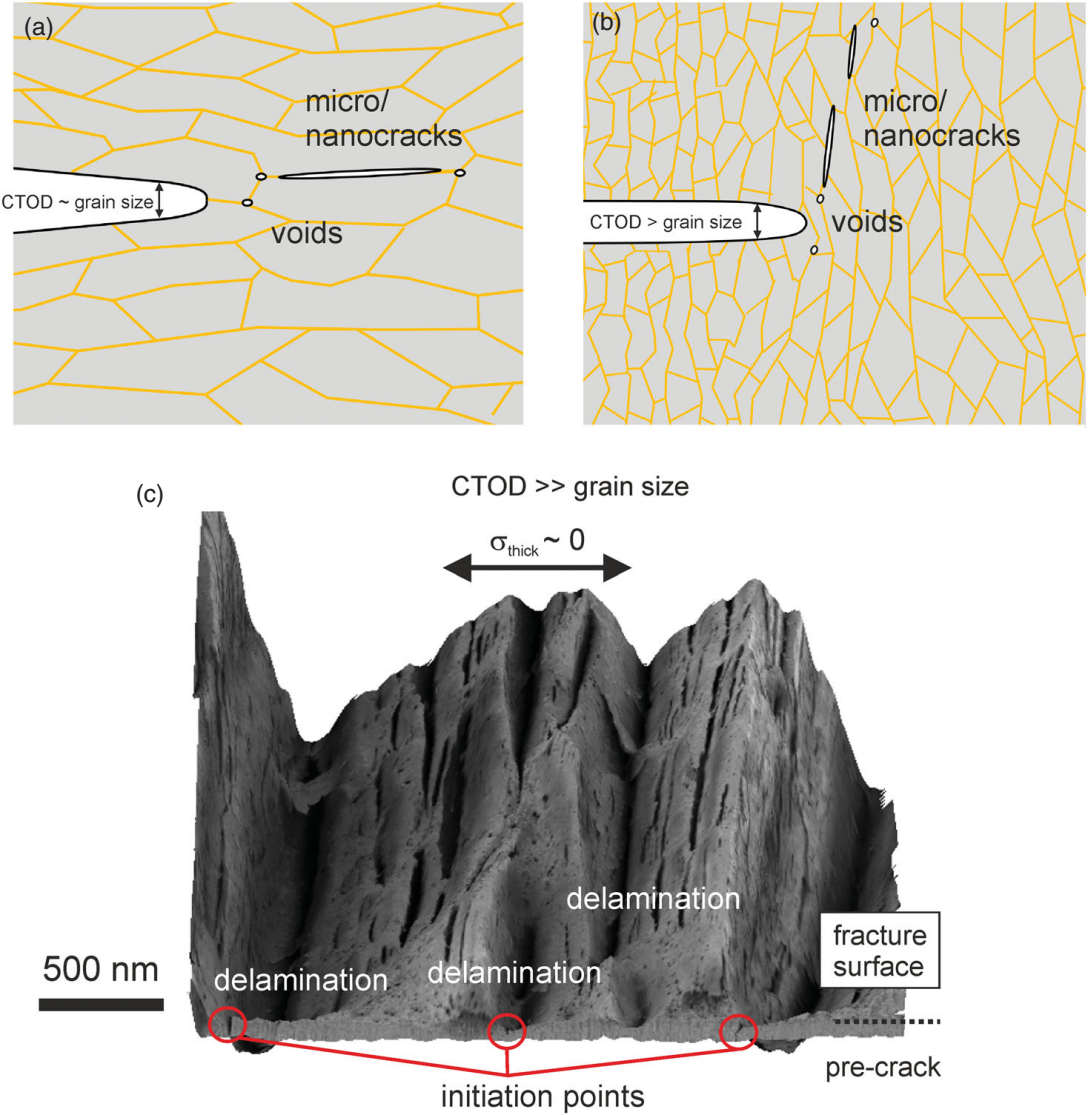
Brittle failure type in NC and UFG metals. (a) Intergranular fracture along the elongated grains. (b) Crack branching into the direction of grain alignment with higher fracture toughness and intergranular fracture. (c) Crack-divider orientation with local crack branching and delamination formation causing a decrease of the through-thickness stress component.

In the second group (brittle type), the crack propagates by grain boundary de-cohesion with intergranular fracture when the crack propagation direction is parallel to the aligned grains, see Figures 5(a) and 2(a), orientation A. The fracture toughness for this loading direction is relatively small. Perpendicular to the long axis of the grains, Figures 5(b) and 2(a), orientation B, the crack propagates also by grain boundary de-cohesion (intergranular) and the fracture toughness is increased. The reason for the toughness increase can be explained with the decrease in the local driving force as a result of the crack deflection.[35] In the last configuration the crack is also oriented perpendicular to a long axis of the grains; however, the grain axis is shorter than in the case described before, also compare with Figure 2(a) orientation C. Here, the crack often propagates by micro-ductile failure combined by delamination and crack branching, see Figure 5(c). The fracture toughness for this loading direction is significantly larger compared to the direction with grain boundary de-cohesion. This can be explained by a decrease in the through-thickness stress component, which reduces the stress-triaxiality when the delaminations form. The crack plane of the initiation points of the delaminations is identical to the crack plane when the crack propagates parallel to the grain alignment, which has a very low fracture toughness. This implies that the delamination formation and so the high fracture toughness is triggered by these weak crack planes.

Despite the general notion that intergranular fracture is associated with pronounced brittleness, significant plastic deformation before and during crack propagation by de-cohesion of the grain boundaries takes place and was proven with CTOD measurements. Hence, plastic bunting of the crack tip occurs before the de-cohesion process starts. The critical crack tip opening displacement is typically equal or somewhat smaller than the small axis of the grain. The anisotropy of the fracture toughness is significantly more pronounced in the second class of materials exhibiting crack propagation by de-cohesion in one loading direction and micro-ductile crack propagation in the other loading direction, which is supported by the formation of delaminations. This behavior has been mainly found in bcc high-strength materials [36,37] such as iron, ferritic steels, tantalum or pearlitic steels.

**Figure 6.**
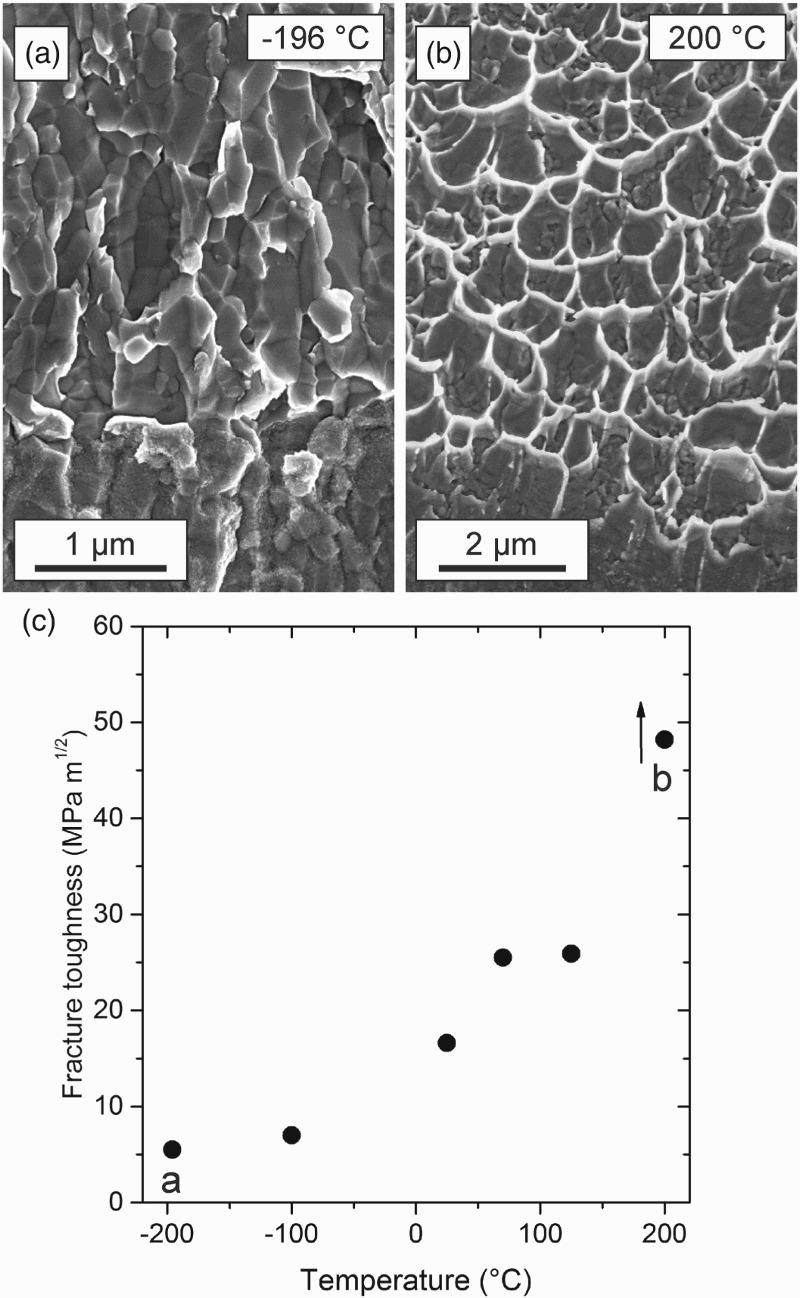
Brittle to ductile transition in SPD-processed iron. (a) Intercrystalline fracture at −196°C. (b) Pure dimple fracture at 200°C. (c) Increase of fracture toughness with increasing temperature with gradual change from grain boundary to dimple fracture.

It is interesting to note that for the loading direction with grain boundary de-cohesion a brittle to ductile transition can be observed, see Figure 6. For example, in SPD-processed iron at low testing temperatures, a grain boundary de-cohesion prevails (Figure 6(a)), whereas for higher temperatures micro-ductile fracture can be found (Figure 6(b)). This gradual change leads to a strong increase of fracture toughness (Figure 6(c)).

The reason for the grain boundary de-cohesion and the intergranular crack path is not fully clear yet. At first, classical grain boundary embrittlement could be a reason for it, however, has not been confirmed yet.[31] Unfortunately this question is difficult to address experimentally. The resulting grain size in the saturation regime during SPD is very sensitive on the impurity content [38] and, therefore, in a systematic study with different purity levels grain size and strength would change as well. Apart from this extrinsic reason, the intergranular crack path could also be an intrinsic feature as proposed by several simulation studies.[39,40]

Even though the presented classification into a brittle and a ductile type is mainly based on material processed by HPT, the same tendencies can also be expected using other SPD processes, such as ECAP or ARB, which all have an elongated microstructure as a common feature. This has already been proven with ECAP-processed iron [36] leading to the same qualitative results as for HPT-processed.[16] Especially in continuous SPD processes, which are more suitable for mass production of UFG and NC structure, such as continuous confined strip shearing [41] or the conshearing process,[42] elongated structures are typical and will control the fracture behavior. In addition to these presented results based on quasi-static experiments, a similar situation has also been found for the cyclic case in terms of crack deviations from the expected crack propagation direction, propagation direction-dependent crack propagation rates and threshold values.[43,44]

## The potential of anisotropy for future material design

**Figure 7.**
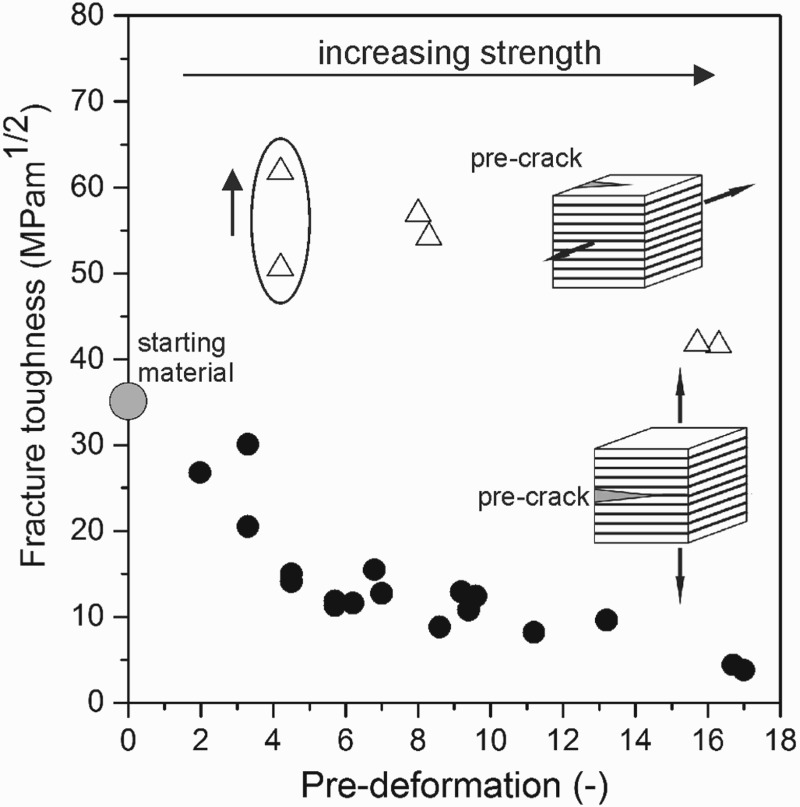
Changes in fracture toughness in a pearlitic steel due to SPD. In the shear orientation (black dots) fracture toughness progressively decreases with increasing alignment of the lamellar structure due to SPD. In the crack-divider orientation with local crack branching and delamination (open triangles) the fracture toughness remains high.

One may assume that this pronounced anisotropy of the fracture toughness is a drawback, especially thinking of the classical engineering viewpoint asking for isotropic mechanical properties. However, in our opinion, this anisotropy represents a promising design concept for future ultra-strong materials with exceptional fracture toughness. Both properties, high fracture toughness and strength, are often combined as one term called damage tolerance. The materials possess one direction with a relatively low fracture toughness but remarkably high fracture toughness for the other loading directions. An example is shown in Figure 7 where the fracture toughness of an HPT-deformed pearlitic steel is shown as a function of pre-deformation in terms of the equivalent Mises strain. With increasing shear strain the lamellae are aligned to the shear direction and the lamellar spacing is reduced from about 200 nm to 20 nm. This induces a huge increase in strength from 900 MPa in the undeformed state to 3,500 MPa [45] at an applied shear strain of *γ* = 30.

The fracture toughness in the crack propagation direction of the aligned microstructure expectedly decreases. However, the fracture toughness in the crack plane orientation perpendicular to the crack plane being aligned parallel to the nano-lamellar structure remains about constant, even though the strength rises with increasing pre-deformation (see also inset images in Figure 7).

This exceptional combination of strength and toughness for this loading direction is a consequence of micro-ductile failure which is supported by delamination cracking. As explained before, these delaminations reduce the tri-axiality in front of the crack and reduce the maximum principle stresses in front of the crack. The maximum principle stress in front of a blunted crack under plane strain condition is about three times the flow stress of a material. Hence, the strength of a tough and ductile material cannot be larger than 1/3 of the theoretical strength. Delaminations, however, reduce the tri-axiality and, therefore, reduce significantly the maximum principle stresses to values close to the flow stress, which means that a stress relaxation takes place. Hence, this delamination process offers the possibility to generate materials with high fracture toughness even for materials and alloys with yield strengths near the theoretical limit. This delamination process is also the reason for the exceptional properties of the new types of cold-drawn pearlitic steels with strength of about 1/3 of the theoretical strength.

For many engineering applications a high toughness is only required in one or two loading directions. Therefore, this anisotropy can be used to overcome this basic discrepancy between strength and toughness (or ductility), in order to obtain an acceptable fracture toughness in the required loading directions. It should be noted that this principle of high strength but anisotropic toughness can also be found in most of our biological materials which have been optimized over millions of years in nature.

## Conclusions

Severely plastically deformed metals often exhibit pronounced anisotropic fracture properties with large variations of the fracture toughness depending on the testing direction. This is a direct consequence of the typically elongated microstructures induced by the majority of SPD processes. The fracture toughness along the elongated microstructure between individual metals can vary strongly as well exhibiting intergranular, transgranular or often a mixed fracture type. These factors make a pure grain-size-dependent description of the fracture toughness to a challenging task. Nevertheless, the described anisotropy should be regarded as a benefit as it allows the creation of highly damaged tolerant materials with exceptional strength levels for well-defined loading cases.

## Note

1. The terms crack-divider and crack-arrestor configuration are often used in fracture mechanics to describe rolled or laminated microstructures in their fracture behavior [23].
